# Body composition and phase angle as an indicator of nutritional status in children with juvenile idiopathic arthritis

**DOI:** 10.1186/s12969-018-0297-y

**Published:** 2018-12-27

**Authors:** Paweł Więch, Izabela Sałacińska, Dariusz Bazaliński, Mariusz Dąbrowski

**Affiliations:** 1 Institute of Nursing and Health Sciences, Faculty of Medicine, University of Rzeszów, Al. mjr. W. Kopisto 2 a, 35-310 Rzeszów, Poland; 2 Diabetic Outpatient Clinic, Medical Center “Beta-Med”, Pl. Wolności 17, 35-073 Rzeszów, Poland

**Keywords:** Bioelectrical impedance, Juvenile idiopathic arthritis, Phase angle, Children, Malnutrition

## Abstract

**Background:**

Juvenile idiopathic arthritis (JIA) is the most common chronic, systemic autoimmune connective tissue disease diagnosed in children and adolescents. An important aspect of monitoring of children with JIA is a precise assessment of the nutritional status to identify children and adolescents at risk of malnutrition. The aim of the study was to assess the body composition and phase angle in children diagnosed with JIA in comparison to age and sex matched healthy children since there are scarce reports in paediatric patients.

**Methods:**

A total of 46 children and adolescents aged 4–18 years, with JIA were included in the cross-sectional study. Controls were selected from the group of healthy children and adolescents. Children with diagnosed JIA and healthy children were strictly matched for age and gender. In both groups BIA with phase angle calculation was performed.

**Results:**

Phase angle score was significantly lower in the study group compared to control group (5.45 ± 0.64 vs. 5.85 ± 0.80, *p* = 0.010). Also lower percentage of body cell mass (50.63 ± 3.46 vs. 52.70 ± 4.06, p = 0.010) and muscle mass (46.02 ± 6.32 vs. 49.53 ± 6.67, *p* = 0.005) were revealed. In the analysis of subtypes of JIA we found significant differences between children and adolescents with polyarthritis compared to control group, while no significant differences were found between patients with oligoarthritis and control group.

**Conclusions:**

The obtained results indicate a higher risk of malnutrition in children and adolescents with JIA compared to healthy peers, predominantly in patients with polyarthritis.

## Background

Juvenile idiopathic arthritis (JIA) is the most common chronic, systemic autoimmune connective tissue disease diagnosed in children and adolescents [1]. An important aspect of monitoring of children with JIA is a precise assessment of the nutritional status to identify children and adolescents at risk of undernourishment. However, the available data in medical databases also indicate the risk of development of overweight and obesity in this group of children. Discrepancy in research in this area probably depends on the subtype of the disease and the pharmacological treatment used [2, 3]. Bioelectrical impedance analysis (BIA) is currently frequently used as a method of body composition assessment, due to ease of use, safety and low cost of this procedure [4, 5]. BIA is the measure of resistance and reactance of the body. The body is made up of both conductive and non-conductive tissues. The conducting tissues are lean tissues with large amount of water and conducting electrolytes. In nonconductive tissues like bone and fat, the fluid content and conducting electrolytes are low. BIA is used indirectly to measure the body fat composition. The total impedance is the total sum of impedance of different tissues. Body fat, total body water and extra cellular water offer electric resistance to electrical current. Cell membranes and tissues interfaces offer capacitive reactance. [6].

To assess the nutritional status of children, regardless of their clinical condition, both fat mass (FM) and fat free mass (FFM) should be measured. The phase angle (PhA) reflects nutritional and functional status of the studied subject and is calculated from the equation:

$$ \mathrm{PhA}=\left(\frac{\mathrm{Xc}}{\mathrm{R}}\right)\mathrm{x}\left(\frac{180^{\mathrm{o}}}{\Pi}\right) $$

where Xc is reactance and R is resistance obtained in BIA [4]. PhAis a linear method of measuring the relationship between electric resistance (R) and reactance (Rc) in series or parallel circuits [6]. Low phase angle score is considered as an indicator of impaired cellular membrane integrity and also indicates the shift of body water from intracellular to extracellular compartments [7]. PhA is an important prognostic factor in numerous disease entities [8, 9]. The European Society for Clinical Nutrition and Metabolism (ESPEN) recommends the assessment of PhA as a prognostic measure of nutrition and a reliable prognostic marker. PhA can be treated as a screening tool to identify patients at risk of deterioration of nutritional status and functionality [10, 11].

The primary aim of the study was to assess the body composition and phase angle in children diagnosed with JIA in comparison to age and sex matched healthy children since there are scarce reports in paediatric patients. We did not find any study assessing both body composition and phase angle in pediatric patients with diagnosed JIA with analysis of subtypes of JIA according to ILAR criteria.

## Methods

### Ethics

The study was approved by the institutional Bioethics Committee at the University of Rzeszów (Resolution No. 5/02/2012) and by all appropriate administrative bodies. The study was conducted in accordance with ethical standards laid down in an appropriate version of the Declaration of Helsinki (as revised in Brazil in 2013) and in Polish national regulations. The study was conducted according to the STROBE criteria.

### Subject

The study group consisted of 46 children diagnosed with JIA, in the Provincial Clinical Hospital No. 2 in Rzeszów at the Pediatric and the Pediatric Neurology Departments. For the purpose of the study, newly diagnosed children and children at various stages of treatment were included. According to the criteria by Ringold and Wallace, an inactive disease phase was indicated in 38 children [12]. Children took only medicines associated with JIA in monotherapy or combination treatment: azathioprine, methotrexate, sulfasalazine, TNF inhibitors, glucocorticosteroids. Due to the combination of more than one drug in many children and hence the inability to allocate to a specific group of drugs, these parameters were not analyzed quantitatively and qualitatively.

Inclusion criteria for the study group were: diagnosed JIA, age of the examined children from 4 to 18 years, lack of other autoimmune or chronic disease that may affect the growth, weight or nutritional status. In addition, legal guardians had to express written consent for children to participate in the study, and adolescents aged 16 and over also had to give their own written consent to participate in the study.

Controls were selected from the group of healthy children and adolescents from primary, middle and high schools from the neighboring urban and rural areas. Children with diagnosed JIA and healthy children were strictly matched for age and gender i.e. every participant with JIA had his/her comparator with the same gender and possibly the nearest date of birth. Detailed characteristics of both groups are presented in the Table 1.

**Table 1:** Demographic and anthropometric parameters of the study participants

Parameter	Juvenile Idiopathic Arthritis (JIA) (*n* = 46) (mean ± SD)	Control for JIA (*n* = 46) (mean ± SD)	*P value*
Age (years)	12.74 ± 3.85	12.70 ± 3.80	0.956
Girls	12.85 ± 3.96	12.79 ± 3.90	0.951
Boys	12.42 ± 3.66	12.42 ± 3.66	1.000
Body weight (kg)	45.39 ± 18.12	46.90 ± 15.26	0.665
Girls	44.84 ± 18.58	45.12 ± 13.08	0.811
Boys	46.93 ± 17.45	51.97 ± 20.05	0.519
Height (cm)	149.33 ± 21.61	153.82 ± 19.07	0.341
Girls	148.03 ± 21.80	153.06 ± 19.02	0.425
Boys	153.00 ± 21.54	155.96 ± 19.91	0.931
BMI (kg/m^2^)	19.42 ± 4.06	19.24 ± 3.06	0.827
Girls	19.40 ± 4.23	18.77 ± 2.65	0.463
Boys	19.47 ± 3.69	20.60 ± 3.80	0.466
Classification systems (ILAR JIA):			
Systemic arthritis	1	n/a	n/a
Oligoarthritis	18	n/a	n/a
Polyarthritis	22	n/a	n/a
Psoriatic arthritis	2	n/a	n/a
Enthesitis-related arthritis	3	n/a	n/a
Undifferentiated arthritis	0	n/a	n/a
Duration of the disease:			
New diagnosis	6	n/a	n/a
More than 6 months	40	n/a	n/a

*SD* Standard deviation, *BMI* Body mass index, *z-score* Standard score, *ILAR JIA* The International League of Associations for Rheumatology classification of juvenile idiopathic arthritis [1]; *n/a* Not applicable

### Assessments

In every study participant, body weight, height were measured and BMI (Body Mass Index) was calculated. Subsequently, bioelectrical impedance analysis with phase angle calculation was performed using the AKERN BIA - 101 analyzer (Akern SRL, Pontassieve, Florence, Italy) to assess nutritional status and body composition. The results were analyzed using dedicated software (Bodygram1_31 from AKERN, Pontassieve, Florence, Italy). Detailed description of methodology was described elsewhere [13].

The parameters of body composition analyzed in BIA included: fat mass (FM), fat free mass (FFM), muscle mass (MM) (kg and %), total body water (TBW), intra- and extra-cellular water (ICW and ECW) (liters and %), body cell mass (BCM) (kg and %) and body cell mass index (BCMI). Upon resistance and reactance results, the phase angle score was calculated. In addition, we also calculated two other indices: fat mass index (FMI) and fat free mass index (FFMI).

### Statistical analysis

Statistical analysis of the data was performed using SigmaPlot for Windows, version 12.5 (Systat Software Inc., San Jose, CA, USA). We performed comparative analysis between two groups as a whole, separate analysis according to gender. Since the most numerous subtypes of JIA were poly- and oligoarthritis, we performed additional comparative analysis between children with these subtypes of JIA and their comparators. The continuous data are presented as mean and SD (standard deviation). Differences between two groups were analyzed using an unpaired two-tailed Student’s t-test after checking normality of distribution by Shapiro-Wilk test and after performing constant variance test. In case of normality and/or constant variance test failure, the Mann-Whitney rank sum test was performed. A *P* value of < 0.05 was considered statistically significant.

## Results

No significant differences in demographic and anthropometric parameters between study and control groups were found (Table 1).

The value of the phase angle in the children with JIA was significantly lower than in the control group of healthy children (*p* = 0.010). The individual parameters of the body composition did not differ statistically between the study group and the control group. Among the children with JIA muscle mass and body cell mass were significantly different in comparison to children from the control group (BCM: *p* = 0.005; MM: p = 0.010) (Table 2).

**Table 2:** Bioelectrical Impedance Analysis results in study participants

Parameter	Juvenile Idiopathic Arthritis (JIA) (*n* = 46) (mean ± SD)	Control (*n* = 46) (mean ± SD)	*P value*
FM (kg)	12.38 ± 6.96	11.08 ± 4.64	0.617
FM (%)	26.62 ± 8.46	23.89 ± 7.58	0.107
FMI	5.34 ± 2.56	4.66 ± 1.89	0.339
FFM (kg)	33.01 ± 13.24	35.82 ± 12.67	0.300
FFM (%)	73.38 ± 8.46	76.11 ± 7.58	0.107
FFMI	14.04 ± 2.48	14.56 ± 2.38	0.310
MM (kg)	20.89 ± 8.96	23.64 ± 9.56	0.175
**MM (%)**	**46.02 ± 6.32**	**49.53 ± 6.67**	**0.005**
TBW (liters)	25.12 ± 9.34	27.10 ± 9.08	0.304
TBW (%)	56.55 ± 7.80	58.00 ± 6.07	0.322
ECW (liters)	11.22 ± 4.48	12.01 ± 3.97	0.295
ECW (%)	44.60 ± 4.21	44.49 ± 2.77	0.585
ICW (liters)	13.89 ± 5.40	15.09 ± 5.25	0.271
ICW (%)	55.40 ± 4.21	55.52 ± 2.77	0.585
BCM (kg)	16.90 ± 7.30	19.21 ± 7.91	0.166
**BCM (%)**	**50.63 ± 3.46**	**52.70 ± 4.06**	**0.010**
BCMI	7.16 ± 1.53	7.76 ± 1.78	0.098
R – resistance (Ohm)	699.57 ± 117.99	656.15 ± 94.83	0.055
X – reactance (Ohm)	66.30 ± 12.19	66.28 ± 7.49	0.784
**PhA**	**5.45 ± 0.64**	**5.85 ± 0.80**	**0.010**

*SD* Standard deviation, *FM* Fat mass, *FMI* Fat mass index, *FFM* Fat free mass, *FFMI* Fat free mass index, *MM* Muscle mass, *TBW* Total body water, *ECW* Extracellular water, *ICW* Intracellular water, *BCM* Body cell mass, *BCMI* Body cell mass index, *PhA* Phase angle. Bold characters indicate significant values (*p*<0.05)

In the analysis according to gender, the phase angle, muscle mass and body cell mass were significantly lower in the girls with JIA compared to control group. In boys, although the differences in these parameters were similar, statistical significance was not reached (Table 3).

**Table 3:** BIA results in JIA participants according to gender

Parameter	Girls		*P value*	Boys		*P value*
	Juvenile Idiopathic Arthritis (JIA), n = 34	Control, *n* = 34		Juvenile Idiopathic Arthritis (JIA), n = 12	Control, *n* = 12	
	Mean ± SD	Mean ± SD		Mean ± SD	Mean ± SD	
FM (kg)	12.81 ± 7.27	11.05 ± 4.16	0.512	11.16 ± 6.12	11.16 ± 6.02	1.000
FM (%)	27.12 ± 6.89	24.34 ± 6.30	0.109	25.20 ± 12.13	22.64 ± 10.67	0.589
FMI	5.45 ± 2.41	4.64 ± 1.60	0.329	5.01 ± 3.04	4.71 ± 2.65	0.840
FFM (kg)	32.03 ± 12.13	34.07 ± 10.06	0.394	35.78 ± 16.25	40.81 ± 17.79	0.386
FFM (%)	72.88 ± 6.89	75.67 ± 6.30	0.109	74.81 ± 12.14	77.36 ± 10.67	0.590
FFMI	13.91 ± 2.30	14.11 ± 1.81	0.702	14.40 ± 3.02	15.84 ± 3.30	0.278
MM (kg)	20.00 ± 8.10	22.05 ± 7.15	0.209	23.43 ± 11.06	28.13 ± 13.81	0.436
**MM (%)**	**44.99 ± 4.39**	**48.54 ± 5.04**	**0.003**	48.95 ± 9.65	52.33 ± 9.70	0.402
TBW (liters)	24.13 ± 8.25	25.68 ± 7.01	0.387	27.91 ± 11.88	31.14 ± 12.87	0.583
TBW (%)	55.73 ± 7.21	57.48 ± 5.32	0.258	58.88 ± 9.22	59.47 ± 7.92	0.868
ECW (liters)	10.82 ± 3.87	11.44 ± 3.20	0.462	12.38 ± 5.94	13.62 ± 5.48	0.453
ECW (%)	44.91 ± 3.97	44.66 ± 2.68	0.762	43.74 ± 4.91	44.00 ± 3.09	0.507
ICW (liters)	13.14 ± 4.68	14.23 ± 3.93	0.270	16.03 ± 6.84	17.53 ± 7.59	0.616
ICW (%)	55.09 ± 3.97	55.34 ± 2.68	0.762	56.26 ± 4.91	56.00 ± 3.09	0.507
BCM (kg)	16.17 ± 6.62	17.89 ± 5.88	0.191	18.98 ± 8.95	22.96 ± 11.46	0.354
**BCM (%)**	**49.87 ± 3.03**	**51.97 ± 3.79**	**0.014**	52.80 ± 3.79	54.77 ± 4.23	0.243
BCMI	6.99 ± 1.42	7.39 ± 1.32	0.235	7.64 ± 1.76	8.81 ± 2.45	0.194
Resistance (ohm)	714.27 ± 104.30	679.82 ± 74.54	0.122	657.92 ± 147.48	589.08 ± 116.11	0.217
Reactance (ohm)	65.85 ± 9.25	67.35 ± 7.71	0.470	67.58 ± 18.67	63.25 ± 6.12	0.840
**PhA**	**5.30 ± 0.53**	**5.70 ± 0.73**	**0.011**	5.87 ± 0.78	6.27 ± 0.88	0.249

*SD* Standard deviation, *FM* Fat mass, *FMI* Fat mass index, *FFM* Fat free mass, *FFMI* Fat free mass index, *MM* Muscle mass, *TBW* Total body water, *ECW* Extracellular water, *ICW* Intracellular water, *BCM* Body cell mass, *BCMI* Body cell mass index, *PhA* Phase angle. Bold characters indicate significant values (*p* < 0.05)

An important finding was observation that in the analysis with regards to the most two prevalent JIA subtypes, significant differences between case and control groups were found only for polyarthritis (lower MM, BCM, BCMI an phase angle) and not oligoarthritis. Despite significantly older age of children with polyarthritis, they had significantly lower percentage of muscle mass compared to children with oligoarthritis (*p* = 0.027). No other significant differences between these groups (also with regards to weight and height) were revealed. We also found a trend towards lower percentage of body cell mass (BCM%), higher FMI and lower PA in children with polyarthritis, compared to group with oligoarthritis, but the level of statistical significance was not attained (Table 4).

**Table 4:** BIA results in JIA participants according to predominant classification systems

Parameter	Oligoarthritis, *n* = 18		Control		*P value*	Polyarthritis, *n* = 22		Control, n = 22		*P value*	*P value* *poly* vs. *oligo*
	Mean	SD	Mean	SD		Mean	SD	Mean	SD		
Age	10.89	4.50	10.89	4.50	1.000	14.00	3.02	13.91	2.93	0.925	**0.037**
Gender (girls/boys)	13/5		13/5		N/A	18/4		18/4		N/A	0.705
BMI	17.86	3.68	19.08	4.15	0.357	19.68	3.59	19.27	2.29	0.655	0.123
FM (kg)	9.57	6.06	10.48	5.78	0.580	13.03	6.96	11.48	3.35	0.673	0.105
FM (%)	24.32	8.65	24.69	8.85	0.950	27.08	7.94	23.46	5.63	0.089	0.299
FMI	4.43	2.23	4.86	2.48	0.537	5.47	2.29	4.53	1.22	0.149	0.077
FFM (kg)	30.31	16.49	31.65	16.03	0.681	33.35	10.29	38.13	8.87	0.113	0.328
FFM (%)	75.69	8.65	75.31	8.85	0.950	72.92	7.94	76.54	5.63	0.089	0.298
FFMI	13.38	2.80	14.21	2.96	0.367	14.18	2.09	14.72	1.91	0.371	0.312
**MM (kg)**	19.67	11.32	20.86	12.22	0.692	**20.69**	**7.09**	**25.17**	**6.51**	**0.035**	0.730
**MM (%)**	48.42	6.98	48.25	6.82	0.943	**44.78**	**5.47**	**50.32**	**5.48**	**0.002**	**0.027**
TBW (liters)	23.00	11.99	24.32	11.52	0.728	25.39	6.70	28.46	6.29	0.110	0.308
TBW (%)	58.12	8.23	58.49	7.27	0.887	56.37	7.59	57.37	4.46	0.445	0.491
ECW (liters)	10.48	5.74	10.48	4.61	0.704	11.28	3.33	12.80	2.88	0.113	0.328
ECW (%)	45.23	3.48	43.84	3.43	0.164	44.45	4.91	44.96	1.98	0.869	0.572
ICW (liters)	12.85	6.93	13.84	7.02	0.635	13.84	3.98	15.66	3.52	0.098	0.399
ICW (%)	54.77	3.48	56.16	3.43	0.164	55.55	4.91	55.04	1.98	0.869	0.572
**BCM (kg)**	15.91	9.20	16.97	10.16	0.716	**16.73**	**5.80**	**20.46**	**5.38**	**0.032**	0.734
**BCM (%)**	51.61	3.64	51.88	4.42	0.841	**49.58**	**3.38**	**53.31**	**3.72**	**0.001**	0.077
**BCMI**	6.97	1.75	7.48	2.20	0.537	**7.07**	**1.35**	**7.91**	**1.38**	**0.049**	0.605
Resistance (ohm)	716.00	134.65	673.56	115.23	0.317	703.14	106.60	648.50	76.66	0.058	0.738
Reactance (ohm)	69.89	13.25	65.94	8.15	0.267	64.36	11.00	67.14	7.41	0.269	0.108
**PhA**	5.63	0.73	5.69	0.89	0.899	**5.26**	**0.58**	**5.96**	**0.72**	**< 0.001**	0.074

*SD* Standard deviation, *FM* Fat mass, *FMI* Fat mass index, *FFM* Fat free mass, *FFMI* Fat free mass index, *MM* Muscle mass, *TBW* Total body water, *ECW* Extracellular water, *ICW* Intracellular water, *BCM* Body cell mass, *BCMI* Body cell mass index, *PhA* Phase angle. Bold characters indicate significant values (p < 0.05)

## Discussion

JIA is the most common systemic disease of autoimmune connective tissue in children and adolescents. Not only joints are affected by the disease but is also characterized by non-articular changes and systemic complications [14, 15].

We did not find significant difference in BMI between study and control groups. Some authors found that patients with JIA have worse nutritional status and lower BMI values compared to healthy children. They explained it as a result of the disease itself and factors such as the inflammatory process, medications and their effects, limited physical activity or increased energy expenditure [14, 16, 17]. Inversely, some other studies indicate higher prevalence of overweight and obesity in affected children [18–20]. Studies in the US demonstrated presence of obesity in 18% of patients with JIA [19]. In Germany as many as 15% of children with JIA were overweight and 7% were obese [20]. Differences between our research and those cited above most often result from the difference in the average age of the examined children or the different activity of the disease.

Analysis of the results with respect to gender showed that the phase angle, muscle mass and body cell mass were significantly lower in girls with JIA. In the group of boys, the size of differences in these parameters was similar, but the statistical significance was not achieved, probably due to the small number of affected boys. Our results confirm the facts, that men have higher phase angles than women due to the higher amount of body muscle mass. Moreover, phase angle increases with increasing BMI due to the increased number of muscle and fat cells. PhA decreases with increasing age, due to a reduction in reactance which parallels the loss of muscle mass and an increase in resistance due to the declining proportion of body water at the expense of increasing fat mass in higher age [4].

Also our patients differences in nutritional status were associated with the JIA subtype, specifically polyarthritis. We found significantly lower percentage of muscle mass in children with polyarthritis compared to patients with oligoarthritis. Furthermore a trend towards lower body cell mass (BCM%), higher FMI and lower PhA in these children was observed. It is in line with other observations. Guzman et al. observed an increase in body mass in various subtypes of JIA in the studied group of children in addition to systemic arthritis [3]. Also Lofthouse et al. in their research pointed out that 18.1% of children with JIA had body mass below the third percentile and patients with polyarticular disease showed significantly more signs of malnutrition than patients with pauciarticular disease. In the polyarticular subtype, in comparison with the control group, they revealed significantly lower body weight, percentage of adipose tissue and total body water [21]. In children with JIA reduced muscle mass and increased fat mass and higher caloric requirements can be noticed. Some researchers recommend bone examination with the assessment of lean mass in this group of children [15, 22, 23].

The prognostic value of our findings should be determined in subsequent studies carried out in this population. ESPEN strongly recommended PhA as a prognostic nutritional measure [24, 25]. The possibility of predicting the occurrence or exacerbation of a given disease is emphasized by many researchers around the world [8, 9, 26, 27]. Hui et al. in their research phase angle retained its prognostic significance in the context of many known prognostic factors. Thus, to being a marker of cellular function, muscle mass and nutritional status, PhA may be a predictive factor of the risk of different complications. Moreover, PhA was weakly but significantly associated with other prognostic variables, suggesting that it captures some additional information compared to existing prognostic factors [26]. There is a promising prognostic tool to use in treatment planning, but further studies (also in JIA) are needed.

One of the most important limitations of the study is the small number of the children with diagnosed JIA included to the study. It did not allow us to demonstrate other significant differences between the study and control groups. In addition, the studied group of children was not analyzed a nutritional status with regards to the pharmacological treatment and disease activity. Nevertheless, our research has shown significant differences in the nutritional status between the study and control groups. Moreover, these differences depended mainly on the subtype of the disease. Also, due to the fact that assessment of important markers of nutritional status (lipids profile, glucose, TSH etc.) was not done in all the children, we were not able to analyze association of these variables with BIA and PhA results. Searching for such relationships would be an intriguing implication for further research.

In the summary, significant differences in body composition and nutritional status between children with JIA and control group indicate the need for implementation of BIA with a phase angle assessment to the routine clinical practice. It can be useful in identifying children at risk of malnutrition in this group of patients, especially among children with polyarthritis.

## Conclusions

The results of this study showed that selected body composition parameters and nutritional indicators, including the phase angle, are lower in children and adolescents with JIA compared to healthy peers. Differences in the nutritional status in the study group depend on the subtype of the disease and children with polyarthritis are at the highest risk of malnutrition. Further prospective studies should be conducted in this group of children to indicate the prognostic value of the results obtained.

## Abbreviations

BCM: Body cell mass
BCMI: Body cell mass index
BIA: Bioelectrical impedance analysis
BMI: Body mass index
ECW: Extra-cellular water
ESPEN: European Society for Clinical Nutrition and Metabolism
FFM: Fat free mass
FFMI: Fat free mass index
FM: Fat mass
FMI: Fat mass index
ICW: Intra-cellular water
ILAR: International League of Associations for Rheumatology
JIA: Juvenile idiopathic arthritis
MM: Muscle mass
PhA: Phase angle
PsA: Psoriatic arthritis
RF: Rheumatoid factor
TBW: Total body water
